# Communicating Scientific Uncertainty About the COVID-19 Pandemic: Online Experimental Study of an Uncertainty-Normalizing Strategy

**DOI:** 10.2196/27832

**Published:** 2021-04-22

**Authors:** Paul K J Han, Elizabeth Scharnetzki, Aaron M Scherer, Alistair Thorpe, Christine Lary, Leo B Waterston, Angela Fagerlin, Nathan F Dieckmann

**Affiliations:** 1 Center for Outcomes Research and Evaluation Maine Medical Center Research Institute Portland, ME United States; 2 Division of General Internal Medicine University of Iowa Carver College of Medicine Iowa City, IA United States; 3 Department of Population Health Sciences University of Utah Salt Lake City, UT United States; 4 Salt Lake City VA Center for Informatics Decision Enhancement and Surveillance Salt Lake City, UT United States; 5 School of Nursing Oregon Health and Science University Portland, OR United States

**Keywords:** uncertainty, communication, ambiguity, vaccination, COVID-19

## Abstract

**Background:**

Communicating scientific uncertainty about public health threats such as COVID-19 is an ethically desirable task endorsed by expert guidelines on crisis communication. However, the communication of scientific uncertainty is challenging because of its potential to promote *ambiguity aversion*—a well-described syndrome of negative psychological responses consisting of heightened risk perceptions, emotional distress, and decision avoidance. Communication strategies that can inform the public about scientific uncertainty while mitigating ambiguity aversion are a critical unmet need.

**Objective:**

This study aimed to evaluate whether an “uncertainty-normalizing” communication strategy—aimed at reinforcing the expected nature of scientific uncertainty about the COVID-19 pandemic—can reduce ambiguity aversion, and to compare its effectiveness to conventional public communication strategies aimed at promoting hope and prosocial values.

**Methods:**

In an online factorial experiment conducted from May to June 2020, a national sample of 1497 US adults read one of five versions of an informational message describing the nature, transmission, prevention, and treatment of COVID-19; the versions varied in level of expressed scientific uncertainty and supplemental focus (ie, uncertainty-normalizing, hope-promoting, and prosocial). Participants then completed measures of cognitive, emotional, and behavioral manifestations of ambiguity aversion (ie, perceived likelihood of getting COVID-19, COVID-19 worry, and intentions for COVID-19 risk-reducing behaviors and vaccination). Analyses assessed (1) the extent to which communicating uncertainty produced ambiguity-averse psychological responses; (2) the comparative effectiveness of uncertainty-normalizing, hope-promoting, and prosocial communication strategies in reducing ambiguity-averse responses; and (3) potential moderators of the effects of alternative uncertainty communication strategies.

**Results:**

The communication of scientific uncertainty about the COVID-19 pandemic increased perceived likelihood of getting COVID-19 and worry about COVID-19, consistent with ambiguity aversion. However, it did not affect intentions for risk-reducing behaviors or vaccination. The uncertainty-normalizing strategy reduced these aversive effects of communicating scientific uncertainty, resulting in levels of both perceived likelihood of getting COVID-19 and worry about COVID-19 that did not differ from the control message that did not communicate uncertainty. In contrast, the hope-promoting and prosocial strategies did not decrease ambiguity-averse responses to scientific uncertainty. Age and political affiliation, respectively, moderated the effects of uncertainty communication strategies on intentions for COVID-19 risk-reducing behaviors and worry about COVID-19.

**Conclusions:**

Communicating scientific uncertainty about the COVID-19 pandemic produces ambiguity-averse cognitive and emotional, but not behavioral, responses among the general public, and an uncertainty-normalizing communication strategy reduces these responses. Normalizing uncertainty may be an effective strategy for mitigating ambiguity aversion in crisis communication efforts. More research is needed to test uncertainty-normalizing communication strategies and to elucidate the factors that moderate their effectiveness.

## Introduction

Public health crises such as the COVID-19 pandemic pose difficult communication challenges, due in large part to the substantial scientific uncertainty surrounding the nature and management of all new and emerging health threats [1]. This uncertainty is a defining feature of public health crises [2,3] and is important to communicate in order to foster public accountability and trust [3-5]. The communication of scientific uncertainty in public health crises is also important because it promotes more realistic expectations about the benefits of risk-reducing actions and allows people to prepare for different potential outcomes. Uncertainty communication in crisis situations has thus been a central focus of expert guidance, such as the Crisis and Emergency Risk Communication (CERC) guidelines issued by the US Centers for Disease Control and Prevention. CERC guidelines recommend that communicators both acknowledge uncertainty by clarifying what is known, what is not known, and what is being done to reduce the uncertainty, and avoid promoting excess certainty about future outcomes that cannot be controlled [6].

The challenge, however, is that uncertainty can have negative psychological effects. As CERC guidelines also acknowledge, the communication of uncertainty can heighten perceptions of risk and promote fear, panic, anxiety, emotional distress, and feelings of hopelessness and helplessness, which can prevent people from taking action [6]. These aversive psychological responses have been empirically documented by a large body of research showing that uncertainty caused by a lack of reliability, credibility, or adequacy of risk information—features of information that constitute what decision theorists have termed *ambiguity* [7]—produces a set of cognitive, emotional, and behavioral responses [8-10]. These include heightened risk perceptions, pessimistic appraisals of risk-reducing actions, fear and anxiety, and avoidance of decision making. These responses, collectively known as *ambiguity aversion*, have been demonstrated in numerous decision-making settings, including health care [7,10,11].

These effects are not universal; individuals vary in their tolerance of ambiguity as well as uncertainty arising from other causes [10-12]. Furthermore, communicating uncertainty can increase, rather than decrease, individuals’ confidence and trust in information when they expect such uncertainty to exist [13-15]. Nevertheless, the predominance of aversive responses to uncertainty for most individuals and situations makes the communication of scientific uncertainty in public health crises challenging [16]. Furthermore, although expert guidelines recommend adjunctive strategies, such as expressing empathy as a means of mitigating the negative psychological effects of uncertainty [5,6], empirical evidence for this or other strategies is lacking, and the optimal methods for communicating uncertainty in public health crises remain unknown [2]. Consequently, available empirical evidence suggests that in these situations scientific uncertainty is rarely communicated in a clear, explicit manner, either by experts or journalists [17-19].

One promising theory-based strategy, however, may be to normalize uncertainty; that is, to emphasize that existing uncertainty is an expected experience that does not indicate an unusual deficit in people’s abilities. A leading theoretical account of ambiguity aversion, the *competence hypothesis*, suggests that ambiguity is aversive because it lowers people’s perceptions of their own competence in decision making [20]. An extension of this account, the *comparative ignorance hypothesis*, posits that aversion to ambiguity about a given prospect is driven by an implicit comparison with a less ambiguous prospect or the state of mind of more knowledgeable individuals [21]. Winkler has posited that ambiguity aversion may also arise from an erroneous belief in the existence of a single ‘‘true’’ objective probability for individual events and a discomfort with not knowing this probability [22]. Together, these theories suggest that ambiguity aversion will be heightened if decision makers’ perceived competence is decreased and their comparative ignorance increased (eg, when they are made aware that relevant risk information is unavailable to them but available to others). In contrast, ambiguity aversion will be diminished if perceived competence is increased and comparative ignorance decreased. Chow and colleagues obtained experimental evidence supporting these effects by showing that individual decision makers’ ambiguity aversion—manifested by their reluctance to bet on an uncertain outcome—diminished when they were made aware that the risks at hand are unknown not only to them but to all individuals; that is, they are *unknowable* [20,23,24].

Normalizing uncertainty as an expected state, therefore, may be a potentially effective strategy for reducing negative psychological responses to the communication of scientific uncertainty in public health crises. The overarching objective of this study was to evaluate this possibility in the real-life context of the COVID-19 pandemic. In a previous experimental study of public responses to uncertainty about a hypothetical viral pandemic [25], we found that an uncertainty-normalizing strategy did not reduce ambiguity-averse cognitive, emotional, and behavioral responses (ie, heightened risk perceptions and worry and diminished vaccination intentions) to scientific uncertainty about the pandemic. However, the generalizability of these findings was limited by the hypothetical nature of the study. In the current study, we addressed this limitation by evaluating the effects of an uncertainty-normalizing strategy in a real public health crisis. Its specific objective was to test whether normalizing uncertainty reduces ambiguity-averse responses, compared to commonly used strategies aimed at promoting either (1) hope or (2) prosocial values [26-28]. These alternative strategies have been broadly implemented in public information campaigns about COVID-19, and focus on mitigating hopelessness, helplessness, and stigmatization—important adverse responses to public health crises [3,5,6]. However, because these strategies do not directly target perceptions of, or responses to, uncertainty, they should be less effective in reducing ambiguity aversion than an uncertainty-normalizing strategy.

To evaluate this possibility, we conducted an online survey–based experiment comparing alternative approaches to communicating scientific uncertainty about multiple aspects of the COVID-19 pandemic, including its controllability, prognosis, and severity. The experiment tested the following hypotheses:

- Hypothesis 1 (H1). The communication of uncertainty about the COVID-19 pandemic will result in ambiguity-averse psychological responses—consisting of greater perceived likelihood of developing COVID-19, greater worry about COVID-19, and lower intentions for COVID-19 risk-reducing behaviors—compared to the noncommunication of uncertainty.
- Hypothesis 2 (H2). Ambiguity-averse responses to the communication of uncertainty about the COVID-19 pandemic will be reduced by uncertainty-normalizing language but not by either hope-promoting or prosocial language.

As an exploratory objective, we also evaluated the extent to which individual differences, including sociodemographic characteristics (ie, age, gender, and education), political affiliation, health literacy, trait-level risk aversion, trait-level ambiguity aversion, and dispositional optimism—all factors that might influence people’s responses to medical uncertainty [11,12,25,29,30]—might moderate the effects of these different uncertainty communication strategies.

## Methods

### Study Design and Experimental Manipulation

The study was part of a larger online experiment, hosted by the internet survey vendor Qualtrics, designed to test different strategies, including language aimed at promoting hope and prosocial values, for communicating to the general public about the nature and prevention of COVID-19. This study focused specifically on strategies for communicating about uncertainty surrounding the COVID-19 pandemic. All alternative strategies were created by adding language to basic information on the nature, transmission, prevention, and treatment of COVID-19, reproduced from a public website produced by a government public health department [31]. This basic information contained no explicit communication of scientific uncertainty and served as the *control* strategy. Supplementing this basic information with additional language resulted in a total of five alternative uncertainty communication strategies, which constituted separate experimental conditions to which participants were randomly assigned: (1) control, (2) uncertainty, (3) uncertainty + uncertainty-normalizing, (4) uncertainty + hope-promoting, and (5) uncertainty + prosocial.

The *uncertainty* condition highlighted the existence of scientific uncertainty about the controllability, prognosis, and severity of the COVID-19 pandemic. The *uncertainty + uncertainty-normalizing* condition combined expressed uncertainty with language emphasizing the unknowability of these various aspects of COVID-19 and the expected nature of scientific uncertainty. The *uncertainty + hope-promoting* condition combined expressed uncertainty with language conveying optimism about future advances in knowledge and control over the pandemic. The *uncertainty + prosocial* condition combined expressed uncertainty with language encouraging awareness of obligations to other community members and concern for the collective good. The alternative uncertainty communication strategies varied in length from 940 to 1273 words; the full text of all strategies is presented in Multimedia Appendix 1.

### Study Population and Recruitment

The study population consisted of a national sample of adult members (aged ≥18 years) of the US public belonging to a voluntary opt-in web survey panel professionally managed by the internet survey vendor Qualtrics. Panel members have experience and interest in completing online surveys for marketing purposes, for which they are provided modest monetary incentives. Qualtrics maintains sociodemographic and geographic data on panel members, which provides the capacity to target recruitment to prespecified quotas in order to achieve a sociodemographically diverse study sample. This study employed quotas aimed at obtaining a balanced distribution by age, gender, race, geographic region of the United States, education level (ie, ≥20% high school diploma or less), and income (ie, ≥50% annual income of US $50,000 or less), and to exclude participants who reported a current or prior diagnosis of COVID-19. To ensure data quality, we excluded participants who gave logically inconsistent responses to two screener questions about participants’ attitudes toward risk-reducing behaviors or whose survey completion time was below 12 minutes—the time cut point accounting for the majority of inconsistent responses in preliminary fielding of the study.

The study was approved by the MaineHealth Institutional Review Board. The survey was fielded from May 7 to June 11, 2020; during this time, the number of total coronavirus infections in the United States increased from >1.2 million to >1.6 million, and total deaths increased from >77,200 to >98,000 [32].

### Measures

After reading their randomly assigned informational vignettes, participants completed a survey questionnaire consisting of the measures summarized below.

#### Outcome Variables

*Perceived uncertainty about COVID-19* served as the manipulation check for the study and was assessed using a 6-item scale (α=.71) developed for this study (see Multimedia Appendix 2). This measure assessed participants’ perceptions of uncertainty arising from various sources (ie, probability, ambiguity, and complexity) and pertaining to various issues (ie, controllability, prognosis, and severity of the COVID-19 pandemic) raised in the experimental vignettes. Example items include “There are conflicting estimates of how long the COVID-19 pandemic will past.” Likert scale response options ranged from 1 (*strongly disagree*) to 7 (*strongly agree*).

*Perceived likelihood of getting COVID-19* was assessed with a single item used in prior studies [25,33,34]: “How likely does it feel that you will get COVID-19 within the next month?” Likert scale response options ranged from 1 (*not at all*) to 7 (*very*).

*Worry about COVID-19* was assessed with a single item used in prior studies [25,33,34]: “How worried are you about getting COVID-19 within the next month?” Likert scale response options ranged from 1 (*not at all*) to 7 (*very*).

*Intentions for COVID-19 risk-reducing behaviors* was assessed by measuring participants’ willingness to follow 14 recommended COVID-19 risk-reducing behaviors (eg, handwashing, avoiding social gatherings, and wearing masks) (see Multimedia Appendix 2). Likert scale response options ranged from 0 (*I am not planning to follow this guideline at all*) to 100 (*I am planning to follow this guideline fully*). Participants’ responses were averaged to create a composite score (α=.95).

*Intentions for vaccination* was assessed with a single item used in prior studies [25,33,34]: “If a vaccine becomes available for COVID-19, how likely would you be to get vaccinated against COVID-19?” Likert scale response options ranged from 1 (*definitely would not get vaccinated*) to 7 (*definitely would get vaccinated*).

#### Covariates and Potential Moderators

*Sociodemographic characteristics* included age (ie, <30, 30-39, 40-49, 50-59, 60-69, and ≥70 years), gender, race, and political affiliation (ie, Democrat, independent or other, and Republican).

*Subjective health literacy* was assessed using an abbreviated, single-item version of a validated health literacy screening measure [35]: “How often do you have someone (like a family member, friend, hospital/clinic worker, or caregiver) help you read instructions, pamphlets, or other written health materials from your doctor or pharmacy?” Likert scale response options ranged from 1 (*never*) to 5 (*always*).

### Data Analysis

**Figure 1:**
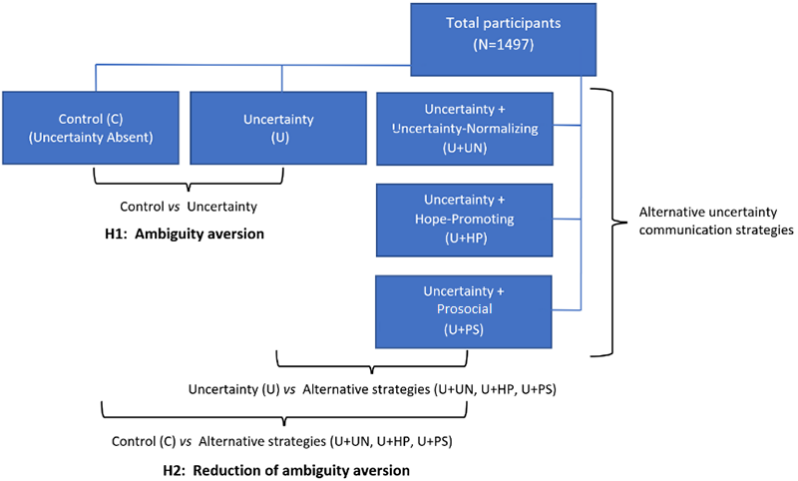
Study design. Alternative uncertainty communication strategies and between-group comparisons. H1: Hypothesis 1; H2: Hypothesis 2.

To compare the effectiveness of alternative uncertainty communication strategies in reducing cognitive, emotional, and behavioral manifestations of ambiguity aversion, we fit analysis of variance models with perceived likelihood of getting COVID-19, worry about COVID-19, and intentions for both risk-reducing behaviors and vaccination as dependent variables and communication strategy as the independent variable. For each dependent variable, we used prespecified contrasts to assess the following: (1) the extent to which the communication of uncertainty produced ambiguity-averse responses, compared to the noncommunication of uncertainty (H1), and (2) the extent to which the three alternative uncertainty communication strategies (ie, uncertainty-normalizing, hope-promoting, prosocial) reduced ambiguity-averse responses (H2) (see Figure 1).

H1 was assessed by the contrast between the *uncertainty* and *control* conditions, while H2 was assessed by contrasts between each of the alternative uncertainty communication strategies and both the *uncertainty* and *control* conditions. For each contrast, we estimated the effect size by calculating Cohen *d*, which represents the standardized mean difference between two groups [36].

To explore potential moderating effects of sociodemographic characteristics (ie, age, gender, race, and political affiliation) and subjective health literacy, we fit separate models with perceived risk of COVID-19, worry about COVID-19, and intentions for both risk-reducing behaviors and vaccination as dependent variables and communication strategy as the independent variable; we entered relevant interaction terms one at a time. All analyses were conducted using SPSS, version 27.0 (IBM Corp).

## Results

### Overview

In our primary conditions, we received data from 1524 respondents. We excluded 2 respondents who gave inconsistent responses and another 25 individuals who reported current or previous COVID-19 illness, leaving a final sample of 1497 respondents (see Table 1). Data were assumed to be missing at random; thus, we utilized a listwise deletion strategy for participants with missing data on any of the outcome measures.

On average, participants took 27.82 (SD 34.59) minutes to complete the study. There were no significant between-group differences in time of completion of the experimental task (*F*_7,2386_=0.479; *P*=.85), suggesting that the cognitive effort required by the task was similar across conditions.

**Table 1:** Sample population characteristics.

Characteristic			Value (N=1497), n (%)
**Age (years)**			
	<30	306 (20.4)	
	30-39	229 (15.3)	
	40-49	209 (14.0)	
	50-59	183 (12.2)	
	60-69	227 (15.2)	
	≥70	343 (22.9)	
**Gender**			
	Male	743 (49.6)	
	Female	748 (50.0)	
	Other or prefer not to say	6 (0.4)	
**Race**			
	White	1003 (67.0)	
	Black or African American	178 (11.9)	
	Asian	147 (9.8)	
	Multiracial or other	169 (11.3)	
**Education**			
	Less than high school	264 (17.6)	
	High school graduate	242 (16.2)	
	Some college or trade school	396 (26.5)	
	College graduate or higher	595 (39.7)	
**Income (US $)**			
	0-24,999	394 (26.3)	
	25,000-49,999	371 (24.8)	
	50,000-99,999	370 (24.7)	
	100,000-149,999	235 (15.7)	
	≥150,000	127 (8.5)	
**Political affiliation**			
	Democrat	540 (36.1)	
	Republican	438 (29.3)	
	Independent or other^a^	519 (34.7)	

^a^Includes other third party or no party affiliation.

### Manipulation Check

Supporting the intended effect of the experimental manipulation, perceived uncertainty about COVID-19 was significantly higher in all experimental conditions containing uncertainty (*F*_4,1492_=3.52; η^2^=0.009; *P*=.007)—that is, *uncertainty* (*d*=–0.28; *P*=.001), *uncertainty + uncertainty-normalizing* (*d*=–0.23; *P*=.006), *uncertainty + hope-promoting* (*d*=–0.20; *P*=.01), and *uncertainty + prosocial* (*d*=–0.22; *P*=.007)—compared to the *control* condition containing no uncertainty. These differences were in the small effect-size range.

### Perceived Likelihood of Getting COVID-19

**Figure 2:**
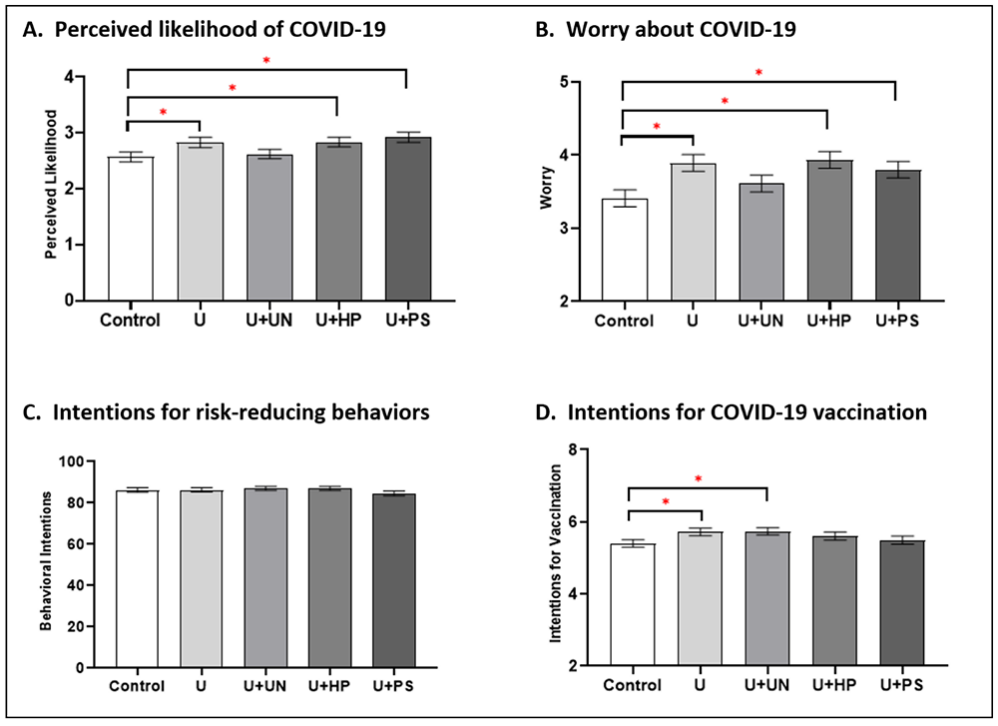
Effects of uncertainty and uncertainty communication strategies on cognitive, emotional, and behavioral manifestations of ambiguity aversion. Asterisks indicate statistically significant pairwise differences (*P*<.05); error bars indicate standard error. U: uncertainty; U+HP: uncertainty + hope-promoting; U+PS: uncertainty + prosocial; U+UN: uncertainty + uncertainty-normalizing.

Consistent with an ambiguity-averse cognitive response to the communication of uncertainty (H1), perceived likelihood of getting COVID-19 was significantly higher in the *uncertainty* condition than in the *control* condition (*F*_4,1492_=2.95; η^2^=0.008; *P*=.02) (see Figure 2A). Supporting the effectiveness of the uncertainty-normalizing strategy in reducing ambiguity aversion (H2), perceived likelihood of getting COVID-19 was not significantly different for the *uncertainty + uncertainty-normalizing* condition compared to the *control* condition (*d*=–0.04; *P*=.66). However, this ambiguity aversion–reducing effect was not seen for the *hope-promoting* (*d*=–0.18; *P*=.03) or *prosocial* (*d*=–0.23; *P*=.005) communication strategies; perceived likelihood remained significantly higher for these conditions than for the *control* condition.

### Worry About COVID-19

Consistent with an ambiguity-averse emotional response to the communication of uncertainty (H1), worry about COVID-19 was significantly higher in the *uncertainty* condition than in the *control* condition (*F*_4,1492_=3.65; η^2^=0.01; *P*=.006) (see Figure 2B). Supporting the effectiveness of the uncertainty-normalizing strategy in reducing ambiguity aversion (H2), worry was not significantly different for the *uncertainty + uncertainty-normalizing* condition compared to the *control* condition (*d*=–0.10; *P*=.21). However, this ambiguity aversion–reducing effect was not seen for the *hope-promoting* (*d*=–0.27; *P*=.001) or *prosocial* (*d*=–0.20; *P*=.02) communication strategies; worry remained significantly higher for these conditions than for the *control* condition.

### Intentions for COVID-19 Risk-Reducing Behaviors and Vaccination

Inconsistent with an ambiguity-averse behavioral response to uncertainty, intentions regarding COVID-19 risk-reducing behaviors (η^2^=0.002; *P*=.49) and vaccination (η^2^=0.005; *P*=.14) showed no significant differences between any of the experimental conditions (see Figure 2C and D). However, prespecified contrasts revealed higher vaccination intentions in both the *uncertainty* (*d*=–0.17; *P*=.04) and the *uncertainty + uncertainty-normalizing* (*d*=–0.18; *P*=.03) conditions compared to the *control* condition, suggesting that the communication of uncertainty itself motivated vaccination intentions and that the addition of uncertainty-normalizing language preserved this motivation (see Figure 2D).

### Moderating Effects

**Figure 3:**
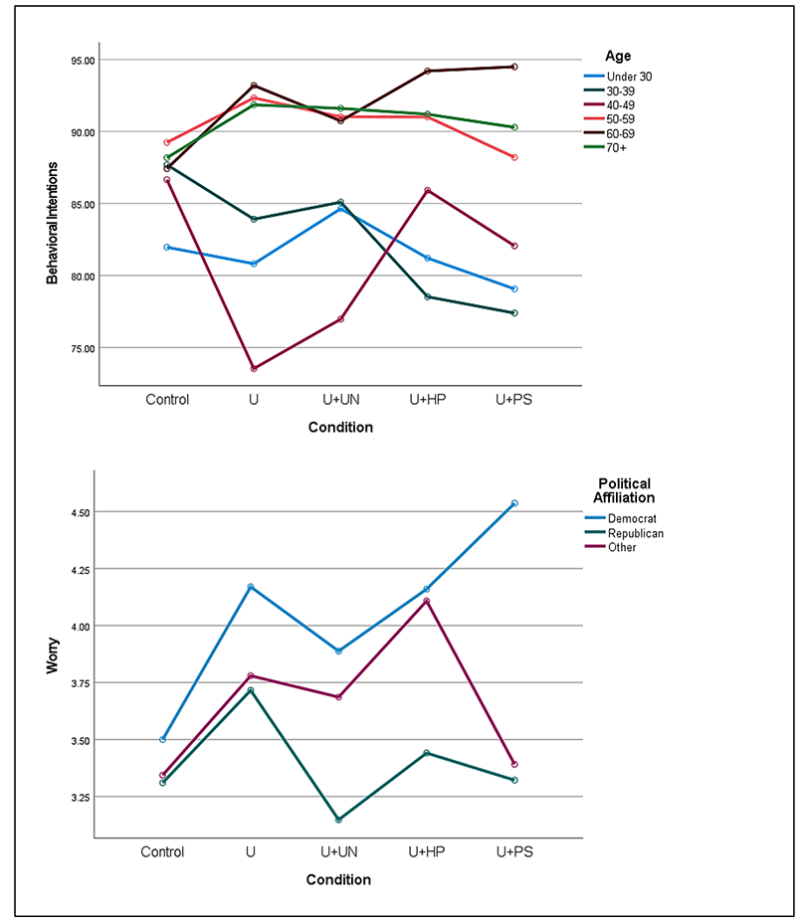
Moderators of the effects of uncertainty communication strategy on manifestations of ambiguity aversion: age and political affiliation. U: uncertainty; U+HP: uncertainty + hope-promoting; U+PS: uncertainty + prosocial; U+UN: uncertainty + uncertainty-normalizing.

Two factors, age and political affiliation, were found to moderate the effects of uncertainty communication strategy on different ambiguity-averse responses to the communication of uncertainty. Age moderated the effect of communication strategy on intentions for COVID-19 risk-reducing behaviors (*F*_20,1445_=1.86; ηρ²=0.025; *P*=.01), such that older participants (ie, aged 50 years and older) generally reported higher intentions in all of the supplementary uncertainty communication conditions compared to the *control* condition, while younger participants (ie, less than 50 years of age) generally reported lower intentions (see Figure 3, top plot).

Political affiliation showed a weaker interaction with uncertainty communication strategy on worry about COVID-19 (ηρ²=0.010; *P*=.06), such that self-reported Republicans had lower worry in the *uncertainty + uncertainty-normalizing* condition compared to the *control* condition, while self-reported Democrats and independents had higher worry (see Figure 3, bottom plot). In other words, the uncertainty-normalizing strategy reduced ambiguity aversion to a greater extent for Republicans than for Democrats. Democrats also had higher worry in the *prosocial* condition compared to the *control* condition, while Republicans and independents had lower worry.

No significant moderating effects were noted for other sociodemographic factors or subjective health literacy.

## Discussion

This experimental study evaluated the comparative effectiveness of different communication strategies in reducing ambiguity-averse cognitive, emotional, and behavioral responses to uncertainty in information about the COVID-19 pandemic. We believe its findings have several implications for future efforts to understand and improve the communication of uncertainty in public health crises.

Consistent with predictions, a strategy aimed at normalizing uncertainty as an expected state of affairs was effective in reducing at least some aversive psychological responses to the communication of uncertainty, whereas widely used alternative strategies aimed at promoting hope and prosocial values had no such effect. A major barrier to open, explicit communication of the uncertainties that inevitably exist during public health crises is a real concern about exacerbating both the perception of vulnerability as well as feelings of fear and panic among the general public [2,3,5,6]. Our findings suggest, however, that language aimed at normalizing these uncertainties can reduce aversive cognitive and emotional responses to them. When uncertainty-normalizing language was added to a message that communicated scientific uncertainty about the COVID-19 pandemic, levels of COVID-19 risk perceptions and worry did not differ from those produced by a message that did not communicate scientific uncertainty. In other words, uncertainty-normalizing language neutralized ambiguity aversion. The overall size of this effect was relatively small, and although uncertainty-normalizing language resulted in lower levels of COVID-19 risk perceptions and worry than those produced by a message that communicated uncertainty alone, this difference was not statistically significant. Nevertheless, even small effects in reducing aversive psychological responses to uncertainty may be beneficial in large-scale efforts to communicate with the general public about health crises. If our findings can be replicated and validated, they suggest a promising new approach to inoculating people against the vulnerability and fear that typically accompany the communication of uncertainty in these situations.

Contrary to predictions, uncertainty-normalizing language had no effect on intentions for COVID-19 risk-reducing behaviors or vaccination. Notably, however, the communication of uncertainty itself also had no effect; it neither decreased nor increased behavioral intentions. In other words, ambiguity aversion in this study was manifest cognitively and emotionally, but not behaviorally. This pattern may be attributable to several factors. Potential negative effects of scientific uncertainty about COVID-19 on intentions for risk-reducing behaviors may have been attenuated by the legally mandated nature of several of these behaviors (eg, mandatory quarantines and regulations requiring social distancing and use of masks). Furthermore, uncertainties about the controllability, prognosis, and severity of the COVID-19 pandemic may have mixed, opposing effects on behavioral intentions. They may decrease intentions by fostering skepticism about the benefits of risk-reducing behaviors, thereby promoting a tendency toward inaction, which is consistent with ambiguity aversion. At the same time, these different uncertainties may also increase intentions by promoting fear about the consequences of avoiding risk-reducing behaviors, thereby promoting a tendency toward action, which is consistent with ambiguity tolerance. More research is needed to understand the factors that moderate people’s behavioral responses to different uncertainties and favor either inaction or action.

Our study sheds light on at least some of these factors. Age moderated the effect of communication strategy on intentions for COVID-19 risk-reducing behaviors. For adults over 50 years of age, all active uncertainty communication strategies resulted in higher behavioral intentions compared to the control (ie, no uncertainty) strategy, while for younger adults, all active uncertainty communication strategies resulted in lower intentions. This moderating effect may be attributable to several factors. Older adults have been identified as being at higher risk for complications of COVID-19 and may, thus, be more motivated to take action in the face of uncertainty. Political affiliation also appeared to partially moderate the effect of communication strategy on worry about COVID-19; the uncertainty-normalizing strategy was more effective in reducing ambiguity aversion for Republicans than for Democrats. This moderating effect is intriguing, and its causes are unclear. Political party affiliation has been shown to influence COVID-19 risk perceptions and intentions for risk-reducing behaviors; Republicans generally demonstrate lower risk perceptions and behavioral intentions than Democrats [37-39]. The differential worry-reducing effect of uncertainty normalization for Republicans versus Democrats suggests the existence of respectively opposing propensities toward either minimizing feelings of vulnerability in the face of uncertainty (for Republicans) or else maximizing them (for Democrats). Political affiliation may be a proxy for numerous factors, including ideologies, values, and worldviews, that may predispose people to more optimistic or pessimistic appraisals of uncertain threats [40]. More research is needed to elucidate how these and other factors produce differential responses to different uncertainty communication strategies and to identify other important moderators.

Our study had several limitations that qualify its findings. The sample consisted of web survey panel members who, by virtue of their willingness to participate regularly in market research and other studies, may not be representative of the general population. However, the sample was large and both geographically and sociodemographically diverse, providing support for the external validity of its findings. Nevertheless, more research is needed to assess the reproducibility of our findings and their generalizability to other populations. The alternative uncertainty communication strategies tested in this study varied modestly in length; however, the absence of significant between-group differences in study completion time suggests that our findings are not attributable solely to differences in cognitive burden or effort. The uncertainty-normalizing language tested in this study was also novel and unvalidated, and it may have contained unintended hope-promoting or prosocial messages. We believe the significant between-group differences observed in our study argue against this possibility; however, further research is needed to ascertain the precision and efficacy of our uncertainty-normalizing language in conveying the normal, expected nature of uncertainty. We also conducted multiple exploratory analyses to identify potential moderators of the effects of different communication strategies; the two significant interactions identified could thus have resulted from chance, and further research is needed to confirm these findings. Finally, some key constructs (ie, perceived uncertainty about COVID-19 and intentions for COVID-19 risk-reducing behaviors) were assessed using new measures that have yet to be validated. Other constructs were assessed using existing single-item measures; however, similar measures have been used in prior studies and shown to have predictive validity [25,30,41-43].

In spite of these limitations, this study yields important new insights on the nature and extent of aversion to ambiguity in the context of the COVID-19 pandemic and on a new and potentially effective uncertainty communication strategy that can minimize this aversion. It remains for future research to confirm our findings and to develop more effective strategies for communicating the unavoidable and irreducible scientific uncertainties that complicate all public health crises.

## Appendix

Multimedia Appendix 1 Experimental conditions.

Multimedia Appendix 2 Measures developed in this study.

## Abbreviations

CERC: Crisis and Emergency Risk Communication
H1: Hypothesis 1
H2: Hypothesis 2
