# Supplementary material for: Communicating Scientific Uncertainty About the COVID-19 Pandemic: Online Experimental Study of an Uncertainty-Normalizing Strategy
Source: J Med Internet Res. 2021 Apr 22;23(4):e27832. doi: 10.2196/27832 (PMC8064708; doi:10.2196/27832)
Supplement: Multimedia Appendix 1 [file jmir_v23i4e27832_app1.docx]

**Appendix 1. Experimental Conditions**

**1. CONTROL (No Uncertainty)**

**2019 Novel Coronavirus (COVID-19)**

This survey is about the 2019 Novel Coronavirus (COVID-19) outbreak, or pandemic—a public health crisis that has affected the whole world and every state in the US. The survey begins with some information about COVID-19. Please take your time and read this information carefully. The survey will then ask you about your attitudes and opinions regarding the COVID-19 pandemic.

**Spread and Symptoms of COVID-19**

COVID-19 is a respiratory illness caused by a coronavirus, a type of virus that infects human beings. It is extremely contagious and spreads from person to person through contact with respiratory secretions from an infected person (e.g., through sneezing and coughing). Anybody can get COVID-19, and nobody is risk-free.

Illness caused by COVID-19 can range from mild to severe. Some people can be infected and have no symptoms at all. More commonly, people with COVID-19 have cough and difficulty breathing, or at least two of these other symptoms: fever, chills, repeated shaking with chills, muscle pain, headache, sore throat, or new loss of taste or smell. Some people have serious illness which can include pneumonia, kidney failure, and death. People who are older and who have other medical problems are at higher risk of dying from COVID-19.

**Prevention and Treatment of COVID-19**

Currently, there is no vaccine that can prevent COVID-19 infection. The only way to prevent COVID-19 and to control the pandemic is to keep it from spreading. Current recommendations include the following:

- Practice good hygiene, including handwashing and use of hand sanitizers
- Practice social distancing (putting space between you and other people)
- Wear a mask
- Self-isolation and self-quarantine: stay home and avoid contact with other people if you’re sick with COVID-19, or have symptoms of it (Self-isolation), or if you may have been exposed to someone with COVID-19 (Self-quarantine)

In addition to these measures, state and local governments in the US have enacted strict sheltering-in-place regulations, travel bans, and “lock-downs” of businesses and schools, in order to limit the spread of the coronavirus.

Currently, there is no effective, widely available treatment that can cure COVID-19 infection. Most people recover at home, without treatment. Some people need to be hospitalized, and require oxygen or breathing machines (mechanical ventilation) to help them recover. But there is no cure for COVID-19 and some people, especially those who are older and who have other medical problems, do not recover.

**2. UNCERTAINTY** (added uncertainty language underlined)

**2019 Novel Coronavirus (COVID-19)**

This survey is about the 2019 Novel Coronavirus (COVID-19) outbreak, or pandemic—a public health crisis that has affected the whole world and every state in the US. The survey begins with some information about COVID-19. Please take your time and read this information carefully. The survey will then ask you about your attitudes and opinions regarding the COVID-19 pandemic.

**Spread and Symptoms of COVID-19**

There are many things we know about COVID-19, and many things we don’t know. COVID-19 is a respiratory illness caused by a coronavirus, a type of virus that infects human beings. It is extremely contagious and spreads from person to person through contact with respiratory secretions from an infected person (e.g., through sneezing and coughing). Anybody can get COVID-19, and nobody is risk-free. It’s impossible to say exactly where it will spread and who will become infected or not.

Illness caused by COVID-19 can range from mild to severe. Some people can be infected and have no symptoms at all. More commonly, people with COVID-19 have cough and difficulty breathing, or at least two of these other symptoms: fever, chills, repeated shaking with chills, muscle pain, headache, sore throat, or new loss of taste or smell. However, not everyone has all of these symptoms, and some people may have additional symptoms other than these. Some people have serious illness which can include pneumonia, kidney failure, and death. People who are older and who have other medical problems are at higher risk of dying from COVID-19. At this time, it’s impossible to predict who will have mild or severe disease.

**Prevention and Treatment of COVID-19**

Currently, there is no vaccine that can prevent COVID-19 infection. Vaccines are under development, but no one knows whether they will be effective. The only way to prevent COVID-19 and to control the pandemic is to keep it from spreading. Current recommendations include the following:

- Practice good hygiene, including handwashing and use of hand sanitizers
- Practice social distancing (putting space between you and other people)
- Wear a mask
- Self-isolation and self-quarantine: stay home and avoid contact with other people if you’re sick with COVID-19, or have symptoms of it (Self-isolation), or if you may have been exposed to someone with COVID-19 (Self-quarantine)

In addition to these measures, state and local governments in the US have enacted strict sheltering-in-place regulations, travel bans, and “lock-downs” of businesses and schools, in order to limit the spread of the coronavirus. No one knows exactly how effective these actions are, but they do help prevent COVID-19 from spreading.

Currently, there is no effective, widely available treatment that can cure COVID-19 infection. Experimental drugs are currently being tested, but so far we don’t know whether they are effective. Most people recover at home, without treatment. Some people need to be hospitalized, and require oxygen or breathing machines (mechanical ventilation) to help them recover. But there is no cure for COVID-19 and some people, especially those who are older and who have other medical problems, do not recover. Unfortunately, it’s not possible to tell for sure whether any single person with COVID-19 will do better or worse.

**Future Management of the COVID-19 Pandemic**

The COVID-19 pandemic is now starting to slow down in several US states and around the world, although the improvement has not been the same across different areas of the US. Nevertheless, lock-downs, strict sheltering-in-place regulations, and social distancing practices have been successful in controlling the spread of the coronavirus, and fewer people are becoming infected with COVID-19 and dying from it. Because of this, several state and city governments are starting to ease or remove these strict measures, and to let people go back to work.

However, there is a risk that the COVID-19 pandemic could get worse again, and cause more suffering and death. Because there still is no vaccine or cure, COVID-19 remains a serious threat that can reappear at any time or place. It can begin spreading again if we stop being careful. Easing lock-downs, opening up businesses and schools, and relaxing social distancing orders could allow the COVID-19 pandemic to get out of control once again, and put people’s lives at risk.

But people disagree about what to do at this point. Right now, no one really knows exactly how long the pandemic will last, how severe it will be, or how effective and necessary lock-downs and strict social distancing measures are. Medical experts and many government officials are urging caution, and believe it is too soon to loosen or remove lock-downs and strict social distancing measures. They believe that until a vaccine is available, COVID-19 will always remain a serious threat to the public’s health. They point out that we don’t know for sure if or when an effective vaccine or treatment will be developed, and until then COVID-19 can always come back again and spread out of control. They also believe that several things are necessary before lock-downs and strict social distancing measures are loosened, including the capability to test many more people for the coronavirus, to isolate people who are infected, and to identify and monitor their close contacts. Right now, it is not clear whether any US state has these capabilities. However, many other government officials do not agree that these things are necessary before loosening lock-downs and strict social distancing measures. They believe it is safe to begin loosening these strict measures right now. State and local governments in the US are therefore taking very different approaches: some are loosening lock-downs and social distancing orders, while others are not. It’s impossible to know for sure, however, which is the right approach.

**3. UNCERTAINTY + UNCERTAINTY-NORMALIZING** (added uncertainty language underlined, uncertainty-normalizing language in bold)

**2019 Novel Coronavirus (COVID-19)**

This survey is about the 2019 Novel Coronavirus (COVID-19) outbreak, or pandemic—a public health crisis that has affected the whole world and every state in the US. The survey begins with some information about COVID-19. Please take your time and read this information carefully. The survey will then ask you about your attitudes and opinions regarding the COVID-19 pandemic.

**Spread and Symptoms of COVID-19**

**Right now,** there are many things we know about COVID-19, and many things we don’t know. COVID-19 is a respiratory illness caused by a coronavirus, a type of virus that infects human beings. It is extremely contagious and spreads from person to person through contact with respiratory secretions from an infected person (e.g., through sneezing and coughing). Anybody can get COVID-19, and nobody is risk-free. It’s impossible to say exactly where it will spread and who will become infected or not. **But this is true of all diseases; there are always risks and the goal of medicine is to reduce these risks.**

**Like most diseases, COVID-19 doesn’t affect everyone in the same way.** Illness caused by COVID-19 can range from mild to severe. Some people can be infected and have no symptoms at all. More commonly, people with COVID-19 have cough and difficulty breathing, or at least two of these other symptoms: fever, chills, repeated shaking with chills, muscle pain, headache, sore throat, or new loss of taste or smell. However, not everyone has all of these symptoms, and some people may have additional symptoms other than these. Some people have serious illness which can include pneumonia, kidney failure, and death. People who are older and who have other medical problems are at higher risk of dying from COVID-19. At this time, it’s impossible to predict who will have mild or severe disease. **As with all diseases in medicine, doctors never really tell which patients will do better or worse.**

**Prevention and Treatment of COVID-19**

Currently, there is no vaccine that can prevent COVID-19 infection. Vaccines are under development, but no one knows whether they will be effective. **It takes time to do the research needed to develop a safe and effective vaccine, so we need to do the best we can until then. For now, t**he only way to prevent COVID-19 and to control the pandemic is to keep it from spreading. Current recommendations include the following:

- Practice good hygiene, including handwashing and use of hand sanitizers
- Practice social distancing (putting space between you and other people)
- Wear a mask
- Self-isolation and self-quarantine: stay home and avoid contact with other people if you’re sick with COVID-19, or have symptoms of it (Self-isolation), or if you may have been exposed to someone with COVID-19 (Self-quarantine)

In addition to these measures, state and local governments in the US have enacted strict sheltering-in-place regulations, travel bans, and “lock-downs” of businesses and schools, in order to limit the spread of the coronavirus. No one knows exactly how effective these actions are, but they do help prevent COVID-19 from spreading. **It’s possible that other measures might also help control the pandemic, but these are the best ones we have right now.**

Currently, there is no effective, widely available treatment that can cure COVID-19 infection. Experimental drugs are currently being tested, but so far we don’t know whether they are effective. **While we wait for medical researchers to find a cure for COVID-19, patients are given the best care possible to help them fight and survive the illness based on what we know about the disease.** Most people recover at home, without treatment. Some people need to be hospitalized, and require oxygen or breathing machines (mechanical ventilation) to help them recover. But there is no cure for COVID-19 and some people, especially those who are older and who have other medical problems, do not recover. Unfortunately, it’s not possible to tell for sure whether any single person with COVID-19 will do better or worse. **As with all problems in medicine, we don’t know everything but we do the best we can with what we know.**

**Future Management of the COVID-19 Pandemic**

The COVID-19 pandemic is now starting to slow down in several US states and around the world, although the improvement has not been the same across different areas of the US. Nevertheless, lock-downs, strict sheltering-in-place regulations, and social distancing practices have been successful in controlling the spread of the coronavirus, and fewer people are becoming infected with COVID-19 and dying from it. Because of this, several state and city governments are starting to ease or remove these strict measures, and to let people go back to work.

However, there is a risk that the COVID-19 pandemic could get worse again, and cause more suffering and death. Because there still is no vaccine or cure, COVID-19 remains a serious threat that can reappear at any time or place. It can begin spreading again if we stop being careful. Easing lock-downs, opening up businesses and schools, and relaxing social distancing orders could allow the COVID-19 pandemic to get out of control once again, and put people’s lives at risk. **We don’t know for sure whether this would happen, because medical knowledge is never good enough to predict the future. The best we can ever do is to act wisely to reduce our risks.**

But people disagree about what to do at this point. Right now, no one really knows exactly how long the pandemic will last, how severe it will be, or how effective and necessary lock-downs and strict social distancing measures are. Medical experts and many government officials are urging caution, and believe it is too soon to loosen or remove lock-downs and strict social distancing measures. They believe that until a vaccine is available, COVID-19 will always remain a serious threat to the public’s health. They point out that we don’t know for sure if or when an effective vaccine or treatment will be developed, and until then COVID-19 can always come back again and spread out of control. They also believe that several things are necessary before lock-downs and strict social distancing measures are loosened, including the capability to test many more people for the coronavirus, to isolate people who are infected, and to identify and monitor their close contacts. Right now, it is not clear whether any US state has these capabilities. However, many other government officials do not agree that these things are necessary before loosening lock-downs and strict social distancing measures. They believe it is safe to begin loosening these strict measures right now. State and local governments in the US are therefore taking very different approaches: some are loosening lock-downs and social distancing orders, while others are not. It’s impossible to know for sure, however, which is the right approach. **It’s normal to disagree about difficult issues such as this, when there’s so much is at stake and so much we don’t know. But our limited knowledge shouldn’t stop us from weighing both sides carefully, and coming up with the best solution we can based on the information we have.**

**4. UNCERTAINTY + HOPE-PROMOTING** (added uncertainty language underlined, hope-promoting language in bold)

**2019 Novel Coronavirus (COVID-19)**

This survey is about the 2019 Novel Coronavirus (COVID-19) outbreak, or pandemic—a public health crisis that has affected the whole world and every state in the US. **We are learning more about it every day**. The survey begins with some information about COVID-19. Please take your time and read this information carefully. The survey will then ask you about your attitudes and opinions regarding the COVID-19 pandemic.

**Spread and Symptoms of COVID-19**

There are many things we know about COVID-19, and many things we don’t know. COVID-19 is a respiratory illness caused by a coronavirus, a type of virus that infects human beings. It is extremely contagious and spreads from person to person through contact with respiratory secretions from an infected person (e.g., through sneezing and coughing). Anybody can get COVID-19, and nobody is risk-free. It’s impossible to say exactly where it will spread and who will become infected or not. **But staying alert and watchful will help your reduce your risk of becoming infected and spreading COVID-19 to others.**

Illness caused by COVID-19 can range from mild to severe. Some people can be infected and have no symptoms at all. More commonly, people with COVID-19 have cough and difficulty breathing, or at least two of these other symptoms: fever, chills, repeated shaking with chills, muscle pain, headache, sore throat, or new loss of taste or smell. However, not everyone has all of these symptoms, and some people may have additional symptoms other than these. Some people have serious illness which can include pneumonia, kidney failure, and death. People who are older and who have other medical problems are at higher risk of dying from COVID-19. At this time, it’s impossible to predict who will have mild or severe disease. **But knowing and looking out for the symptoms will protect yourself and the people around you**.

**Prevention and Treatment of COVID-19**

Currently, there is no vaccine that can prevent COVID-19 infection. Vaccines are under development, but no one knows whether they will be effective. **However, scientists are working on developing and testing different possible vaccines, and are optimistic that an effective vaccine will be available some time in the next year. In the meantime, t**he only way to prevent COVID-19 and to control the pandemic is to keep it from spreading, **and there are many things we can do**. Current recommendations include the following:

- Practice good hygiene, including handwashing and use of hand sanitizers
- Practice social distancing (putting space between you and other people)
- Wear a mask
- Self-isolation and self-quarantine: stay home and avoid contact with other people if you’re sick with COVID-19, or have symptoms of it (Self-isolation), or if you may have been exposed to someone with COVID-19 (Self-quarantine)

In addition to these measures, state and local governments in the US have enacted strict sheltering-in-place regulations, travel bans, and “lock-downs” of businesses and schools, in order to limit the spread of the coronavirus. No one knows exactly how effective these actions are, but they do help prevent COVID-19 from spreading. **Taking care and doing all you can to follow these recommendations will keep you and everyone around you safer.**

Currently, there is no effective, widely available treatment that can cure COVID-19 infection. Experimental drugs are currently being tested, but so far we don’t know whether they are effective. **However, the research is promising, and scientists are hopeful that they will find an effective treatment soon.** Most people recover at home, without treatment. Some people need to be hospitalized, and require oxygen or breathing machines (mechanical ventilation) to help them recover. But there is no cure for COVID-19 and some people, especially those who are older and who have other medical problems, do not recover. Unfortunately, it’s not possible to tell for sure whether any single person with COVID-19 will do better or worse. **But good medical care will help many people make it through the infection**.

**Future Management of the COVID-19 Pandemic**

The COVID-19 pandemic is now starting to slow down in several US states and around the world, although the improvement has not been the same across different areas of the US. Nevertheless, lock-downs, strict sheltering-in-place regulations, and social distancing practices have been successful in controlling the spread of the coronavirus, and fewer people are becoming infected with COVID-19 and dying from it. **These are very encouraging signs that our hard work is paying off, and we are gaining control of this problem and making it through the crisis.** Because of this, several state and city governments are starting to ease or remove these strict measures, and to let people go back to work.

However, there is a risk that the COVID-19 pandemic could get worse again, and cause more suffering and death. Because there still is no vaccine or cure, COVID-19 remains a serious threat that can reappear at any time or place. It can begin spreading again if we stop being careful. Easing lock-downs, opening up businesses and schools, and relaxing social distancing orders could allow the COVID-19 pandemic to get out of control once again, and put people’s lives at risk.

But people disagree about what to do at this point. Right now, no one really knows exactly how long the pandemic will last, how severe it will be, or how effective and necessary lock-downs and strict social distancing measures are. Medical experts and many government officials are urging caution, and believe it is too soon to loosen or remove lock-downs and strict social distancing measures. They believe that until a vaccine is available, COVID-19 will always remain a serious threat to the public’s health. They point out that we don’t know for sure if or when an effective vaccine or treatment will be developed, and until then COVID-19 can always come back again and spread out of control. They also believe that several things are necessary before lock-downs and strict social distancing measures are loosened, including the capability to test many more people for the coronavirus, to isolate people who are infected, and to identify and monitor their close contacts. Right now, it is not clear whether any US state has these capabilities. However, many other government officials do not agree that these things are necessary before loosening lock-downs and strict social distancing measures. They believe it is safe to begin loosening these strict measures right now. State and local governments in the US are therefore taking very different approaches: some are loosening lock-downs and social distancing orders, while others are not. It’s impossible to know for sure, however, which is the right approach. **Regardless of what course we choose, however, we need to do our very best to reduce the threat of COVID-19. This means staying cautious and not letting up but keeping up our efforts to prevent, detect, and treat this deadly disease, until this disease is eliminated. If we continue to work hard to protect ourselves, we will make it through this crisis, stronger than we were before.**

**5. UNCERTAINTY + PRO-SOCIAL** (added uncertainty language underlined, pro-social language in bold)

**2019 Novel Coronavirus (COVID-19)**

This survey is about the 2019 Novel Coronavirus (COVID-19) outbreak, or pandemic—a public health crisis that has affected the whole world and every state in the US. **During this time of crisis in our country and our world, our connection to one another become more important than ever. Relationships with our families, our loved ones, and our communities provide us with courage and support, and it is through our connection with others that we are able to navigate tough situations. Please take a moment and think about what the connections in your life mean to you.** The survey begins with some information about COVID-19. Please take your time and read this information carefully. The survey will then ask you about your attitudes and opinions regarding the COVID-19 pandemic.

**The COVID-19 Pandemic**

**To protect our country from the COVID-19 pandemic, we all need to put aside our differences and join together. It is important that we not only protect ourselves, but also one another, including our families, loved ones, neighbors, and our communities. Our health is one of the most important things we have in life, and by working together, we can preserve our health and make our world a safer place. We’re all in this together.**

**Spread and Symptoms of COVID-19**

**Knowing how COVID-19 is spread can help you stay healthy, and reduce the risk of infection for you, your loved ones, and other people in your community. COVID-19 is no one’s fault, but everyone’s responsibility.**

There are many things we know about COVID-19, and many things we don’t know. COVID-19 is a respiratory illness caused by a coronavirus, a type of virus that infects human beings. It is extremely contagious and spreads from person to person through contact with respiratory secretions from an infected person (e.g., through sneezing and coughing). Anybody can get COVID-19, and nobody is risk-free. It’s impossible to say exactly where it will spread and who will become infected or not. **That means we all need to prevent spreading the COVID-19 to each other.**

Illness caused by COVID-19 can range from mild to severe. Some people can be infected and have no symptoms at all. More commonly, people with COVID-19 have cough and difficulty breathing, or at least two of these other symptoms: fever, chills, repeated shaking with chills, muscle pain, headache, sore throat, or new loss of taste or smell. However, not everyone has all of these symptoms, and some people may have additional symptoms other than these. Some people have serious illness which can include pneumonia, kidney failure, and death. People who are older and who have other medical problems are at higher risk of dying from COVID-19. At this time, it’s impossible to predict who will have mild or severe disease. **We need to take special care to protect our family members and loved ones who belong to these groups.**

**Prevention and Treatment of COVID-19**

Currently, there is no vaccine that can prevent COVID-19 infection. Vaccines are under development, but no one knows whether they will be effective. The only way to prevent COVID-19 and to control the pandemic is to keep it from spreading. **Your actions can make the world safer for your loved ones and your community.** Current recommendations include the following:

- Practice good hygiene, including handwashing and use of hand sanitizers
- Practice social distancing (putting space between you and other people)
- Wear a mask
- Self-isolation and self-quarantine: stay home and avoid contact with other people if you’re sick with COVID-19, or have symptoms of it (Self-isolation), or if you may have been exposed to someone with COVID-19 (Self-quarantine)

**Following these recommendations can protect you, your loved ones, and your community.** In addition to these measures, state and local governments in the US have enacted strict sheltering-in-place regulations, travel bans, and “lock-downs” of businesses and schools, in order to limit the spread of the coronavirus. No one knows exactly how effective these actions are, but they do help prevent COVID-19 from spreading. **These measures require us all to share responsibility for controlling COVID-19. Because it can infect anyone, everyone needs to work together to prevent it**.

Currently, there is no effective, widely available treatment that can cure COVID-19 infection. Experimental drugs are currently being tested, but so far we don’t know whether they are effective. Most people recover at home, without treatment. Some people need to be hospitalized, and require oxygen or breathing machines (mechanical ventilation) to help them recover. But there is no cure for COVID-19 and some people, especially those who are older and who have other medical problems, do not recover. Unfortunately, it’s not possible to tell for sure whether any single person with COVID-19 will do better or worse. **People infected with COVID-19 should seek medical attention, so that they can receive the best care possible for the illness.**

**Future Management of the COVID-19 Pandemic**

The COVID-19 pandemic is now starting to slow down in several US states and around the world, although the improvement has not been the same across different areas of the US. Nevertheless, lock-downs, strict sheltering-in-place regulations, and social distancing practices have been successful in controlling the spread of the coronavirus, and fewer people are becoming infected with COVID-19 and dying from it. **This success is showing that if we work together, we can keep each other and our communities safe and healthy.** Because of this, several state and city governments are starting to ease or remove these strict measures, and to let people go back to work.

However, there is a risk that the COVID-19 pandemic could get worse again, and cause more suffering and death. Because there still is no vaccine or cure, COVID-19 remains a serious threat that can reappear at any time or place. It can begin spreading again if we stop being careful. Easing lock-downs, opening up businesses and schools, and relaxing social distancing orders could allow the COVID-19 pandemic to get out of control once again, and put people’s lives at risk.

But people disagree about what to do at this point. Right now, no one really knows exactly how long the pandemic will last, how severe it will be, or how effective and necessary lock-downs and strict social distancing measures are. Medical experts and many government officials are urging caution, and believe it is too soon to loosen or remove lock-downs and strict social distancing measures. They believe that until a vaccine is available, COVID-19 will always remain a serious threat to the public’s health. They point out that we don’t know for sure if or when an effective vaccine or treatment will be developed, and until then COVID-19 can always come back again and spread out of control. They also believe that several things are necessary before lock-downs and strict social distancing measures are loosened, including the capability to test many more people for the coronavirus, to isolate people who are infected, and to identify and monitor their close contacts. Right now, it is not clear whether any US state has these capabilities. However, many other government officials do not agree that these things are necessary before loosening lock-downs and strict social distancing measures. They believe it is safe to begin loosening these strict measures right now. State and local governments in the US are therefore taking very different approaches: some are loosening lock-downs and social distancing orders, while others are not. It’s impossible to know for sure, however, which is the right approach. **Regardless of what course we choose, however, if we continue to care for each other and our communities, we will control the COVID-19 pandemic. We’re all in this together.**
