# Supplementary material for: Communicating Scientific Uncertainty About the COVID-19 Pandemic: Online Experimental Study of an Uncertainty-Normalizing Strategy
Source: J Med Internet Res. 2021 Apr 22;23(4):e27832. doi: 10.2196/27832 (PMC8064708; doi:10.2196/27832)
Supplement: Multimedia Appendix 2 [file jmir_v23i4e27832_app2.docx]

**Appendix 2. Measures developed in this study**

A. Perceived uncertainty about COVID-19

Please rate your agreement with the following statements. There are no right or wrong answers.

1. Whether or not the COVID-19 pandemic can be controlled is mostly a matter of chance.
2. There are conflicting recommendations about how to control the COVID-19 pandemic.
3. Controlling the COVID-19 pandemic is really complicated.
4. How long the COVID-19 pandemic will last is mostly a matter of chance.
5. There are conflicting estimates of how long the COVID-19 pandemic will last.
6. Predicting how long the COVID-19 pandemic will last is really complicated.

_____________________________________________________________________________________

7-point response scale, ranging from Strongly Disagree (1) to Strongly Agree (7)

B. Intentions for COVID-19 risk-reducing behaviors

How fully do you plan to follow each of these guidelines over the next month? Please use the slider bar to indicate your answer, using the response scale below:

0 10 20 30 40 50 60 70 80 90 100

I am NOT planning I AM planning

to follow this to follow this

guideline AT ALL guideline FULLY

1. Avoid gatherings of 10 or more people
2. If someone in your house has tested positive, keep the entire household at home. Do not go to work or school.
3. Avoid optional travel.
4. Avoid optional shopping trips.
5. Avoid optional social visits.
6. Avoid eating or drinking in restaurants, bars, & food courts. Use drive-though, pickup, and delivery options.
7. Do not visit nursing homes or retirement or long-term care facilities unless to provide critical assistance.
8. Practice good hygiene, such as washing your hands, especially after touching frequently used items or surfaces. Avoid touching your face.
9. Keeping a safe distance of at least 6 feet from other people.
10. Wear a mask covering your nose and mouth.
11. Self-isolating if you have symptoms of COVID-19.
12. Self-quarantining if you have been exposed to someone with a known COVID-19 infection, or have been traveling to another country or area identified as a COVID-19 hot spot.
13. Calling your health care provider if you develop symptoms including cough, fever, or difficulty breathing.
14. Practice strict sheltering-in-place (staying at home, away from work and school)
